# Immunotherapeutic Effects of Different Doses of *Mycobacterium tuberculosis ag85a/b* DNA Vaccine Delivered by Electroporation

**DOI:** 10.3389/fimmu.2022.876579

**Published:** 2022-05-04

**Authors:** Yan Liang, Lei Cui, Li Xiao, Xiao Liu, Yourong Yang, Yanbo Ling, Tong Wang, Lan Wang, Jie Wang, Xueqiong Wu

**Affiliations:** Tuberculosis Prevention and Control Key Laboratory, Beijing Key Laboratory of New Techniques of Tuberculosis Diagnosis and Treatment, Senior Department of Tuberculosis, The Eighth Medical Center of PLA General Hospital, Beijing, China

**Keywords:** DNA vaccine, ag85a/b DNA, immunotherapy, *Mycobacterium tuberculosis*, electroporation

## Abstract

**Background:**

Tuberculosis (TB) is a major global public health problem. New treatment methods on TB are urgently demanded.

**Methods:**

Ninety-six female BALB/c mice were challenged with 2×10^4^ colony-forming units (CFUs) of MTB H_37_Rv through tail vein injection, then was treated with 10μg, 50μg, 100μg, and 200μg of *Mycobacterium tuberculosis* (MTB) *ag85a/b* chimeric DNA vaccine delivered by intramuscular injection (IM) and electroporation (EP), respectively. The immunotherapeutic effects were evaluated immunologically, bacteriologically, and pathologically.

**Results:**

Compared with the phosphate-buffered saline (PBS) group, the CD4^+^IFN-γ^+^ T cells% in whole blood from 200 µg DNA IM group and four DNA EP groups increased significantly (*P*<0.05), CD8^+^IFN-γ^+^ T cells% (in 200 μg DNA EP group), CD4^+^IL-4^+^ T cells% (50 μg DNA IM group) and CD8^+^IL-4^+^ T cells% (50 μg and 100 μg DNA IM group, 100 μg and 200 μg DNA EP group) increased significantly only in a few DNA groups (*P*< 0.05). The CD4^+^CD25^+^ Treg cells% decreased significantly in all DNA vaccine groups (*P*<0.01). Except for the 10 μg DNA IM group, the lung and spleen colony-forming units (CFUs) of the other seven DNA immunization groups decreased significantly (*P*<0.001, *P*<0.01), especially the 100 μg DNA IM group and 50 μg DNA EP group significantly reduced the pulmonary bacterial loads and lung lesions than the other DNA groups.

**Conclusions:**

An MTB *ag85a/b* chimeric DNA vaccine could induce Th1-type cellular immune reactions. DNA immunization by EP could improve the immunogenicity of the low-dose DNA vaccine, reduce DNA dose, and produce good immunotherapeutic effects on the mouse TB model, to provide the basis for the future human clinical trial of MTB *ag85a/b* chimeric DNA vaccine.

## Introduction

Tuberculosis (TB) remains one of the major infectious diseases leading to death. In 2021, there were 10 million incident cases and 1.28 million deaths (1). The epidemic of TB presents a new challenge to the control of tuberculosis. *Mycobacterium bovis* Bacillus Calmette-Guérin (BCG) is an effective vaccine protecting from TB in childhood, but its efficacy against adult pulmonary TB remains controversial (2). Thus, new, safe, and effective TB vaccines are needed. DNA vaccine, one of the latest biotechnological breakthroughs, is the beginning of a new chapter in the vaccine field. DNA vaccines have performed well in clinical trials for diseases such as cancer, human acquired immunodeficiency syndrome (AIDS), malaria, hepatitis B, influenza, allergies, and autoimmunity diseases, demonstrating their potential for disease prevention and immunotherapy (3, 4). DNA vaccines have some advantages, for example, they are more stable, cost-efficient, safe in handling, and easy to produce, transport and store. They can induce comprehensive immune responses including antibody immunity, helper T cell immunity, cytotoxic T cell immunity (5). Although DNA vaccine has strong immunogenicity in small animal models, DNA vaccine has poor performance in large animal and human clinical trials, especially the results in human trials are regret, and it requires very high doses, which creates a barrier to its clinical application (6–10). The main reason for the relatively low immunogenicity of DNA vaccines in large animals and humans is that the amount of antigen expressed by plasmid DNA is insufficient to induce a strong immune responses. Therefore, enhancing the immune effect of DNA vaccine has become a hotspot in this field (11, 12). Strategies for optimizing DNA vaccines include carrier and gene optimization, new adjuvants, and more efficient gene delivery pathways (13–17). In recent years, the development of vaccine delivery technologies such as gene gun, liposome, microparticle, nanoparticle, and electroporation (EP) *in vivo* has significantly improved the delivery efficiency of DNA vaccine (15–19). *In vivo* EP, as an effective route of DNA vaccine delivery, can significantly enhance the immune effect of DNA vaccine for cancer, AIDS, malaria, hepatitis B, influenza, Ebola, dengue fever, and autoimmunity diseases in small animals, even in large animals, including humans (10, 12, 20–22). It has the advantages of high transfection efficiency, simple operation, strong repeatability, high safety, and wide application for mammalian *in vivo* gene transfer (11). Under the action of the electric field, a “hole” is opened instantly on the cell membrane, which can improve the probability of the target gene entering the cell and thus improve the expression efficiency. The slight tissue damage caused by EP also promotes the recruitment of pro-inflammatory cells to the inoculation site and induces the local secretion of cytokines and lymphocyte infiltration to improve antigen presentation and enhance the body’s immune responses (23–28).

In previous studies, the *Mycobacterium tuberculosis* (MTB) *ag85a/b* DNA vaccine had good immunotherapeutic effects (29). At present, *in vivo* EP, as an effective route of TB DNA vaccine delivery, is in the pre-clinical research stage. Our research and others had shown that EP was a safe, effective, and non-toxic side effect, which can significantly enhance the immune effect of DNA vaccine (30–34). Although the IM/EP delivery method has been applied to various disease models, anti-TB immunity mainly depends on cellular immune responses, the effect of IM/EP delivery on the dose-efficacy relationship of the tuberculosis DNA vaccine will also be different from other diseases. Therefore, in this study, we firstly used intramuscular injection (IM) and EP technology to deliver different doses of the MTB *ag85a/b* DNA vaccine and compared their immunotherapeutic effects on the mouse TB model, to provide a basis for improving the immunotherapeutic effects of MTB *ag85a/b* DNA vaccine in future human clinical trials and reducing the dosage of DNA vaccine.

## Materials And Methods

### Ethics Statement

All of the experiments involving animals were approved and conducted by the Animal Ethical Committee of the Eighth Medical Center of the Chinese PLA General Hospital. Mouse care met the standards of the Experimental Animal Regulation Ordinances defined by the China National Science and Technology Commission.

### Mice

Ninety-six 6-8 week age of the specific pathogen-free (SPF) female BALB/c mice were purchased from Beijing Vital River Laboratory Animal Technology Company Limited, China, maintained under infection barrier conditions in a negative pressure animal room in the Eighth Medical Center of the Chinese PLA General Hospital, Beijing, China. They fed a sterile commercial mouse diet (Beijing KeAoXieLi Company Limited, China).

### MTB Strain

MTB H_37_Rv was provided by National Institutes for Food and Drug Control, Beijing, China.

The MTB H_37_Rv strain was inoculated on a Lowenstein-Jensen medium (Baso Biotechnology Co., LTD., Zhuhai, Guangdong province, China) and incubated at 37°C for three weeks. Bacterial colonies were collected, weighed, and homogenized in 0.5% Tween 80-saline, then 0.1mg/ml bacterial suspension was prepared and stored at -20°C. Before use, the frozen bacterial suspension was thawed, diluted to 0.5mg/ml, and then further serially diluted 10-fold. 0.1 ml of each dilution was plated in duplicate onto Lowenstein-Jensen plates and incubated at 37°C for 4 weeks. The MTB colonies on each plate were enumerated. The 0.05mg/ml MTB H_37_Rv suspension contained 5×10^4^ CFUs/ml, each mouse was injected with 0.4 ml through tail vein. In this study, one mouse was challenged with 2×10^4^ CFUs of MTB H_37_Rv through tail vein injection.

### MTB *ag85a/b* Chimeric DNA and Recombinant Ag85AB Chimeric Protein

The method of constructing MTB *ag85a/b* chimeric DNA was described previously (29). Briefly, the DNA encoding amino acids 125-282 in the Ag85B protein of MTB H_37_Rv were amplified by PCR with specific oligonucleotide primers containing the site of endonuclease enzyme Acc I. PCR product was purified and then inserted into the endonuclease enzyme Acc I site of the *ag85a* gene that was cloned into eukaryotic expression vector pVAX1 (Invitrogen Life Technologies Corporation, Carlsbad, CA, USA). The length of gene sequence encoding for strong immunogen Ag85A was 888 bp, and the Ag85B was 474 bp. MTB *ag85a/b* chimeric DNA vaccine was thus constructed by inserting the sequence encoding amino acids 125-282 of Ag85B protein into nucleotide 430-435 (endonuclease enzyme Acc I site) of Ag85A DNA vaccine. In this study, the MTB *ag85a/b* chimeric DNA was produced and purified by Guangzhou Baiyunhan Baidi Biological Medicine Co.Ltd (Guangdong province, China).

### Treatment of MTB-Infected Mice

**Figure 1:**
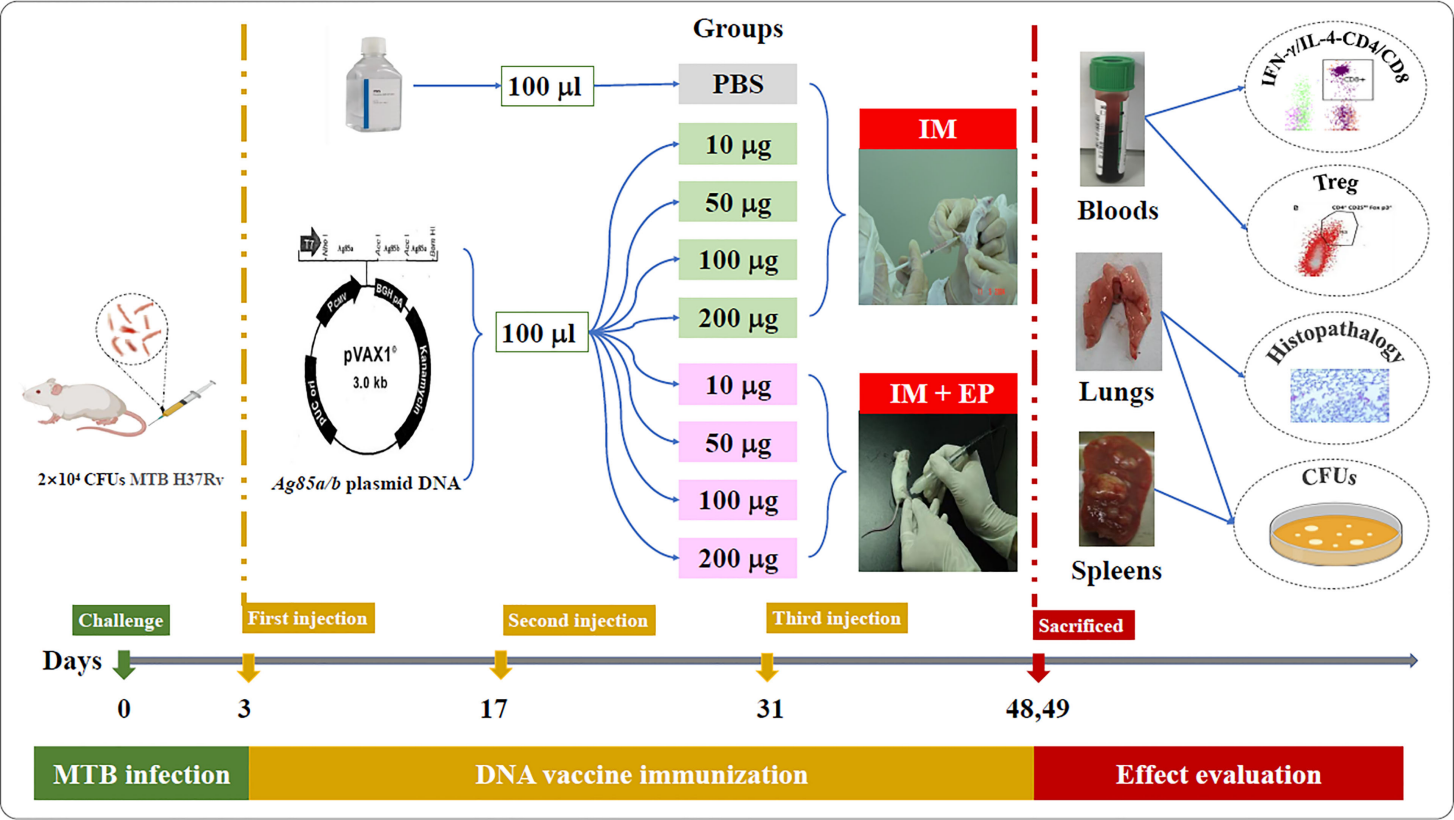
The flow chart of DNA vaccine treatment on the mouse TB model. IM, intramuscular injection; EP, electroporation.

The flow chart of DNA vaccine treatment on the mouse TB model is shown in **Figure 1**. Ninety-six female BALB/c mice were challenged with 2×10^4^ colony-forming units (CFUs) of MTB H_37_Rv through tail vein injection, randomly divided into nine groups as follow: (1) PBS as a negative control (100 μl); (2) 10 μg *ag85a/b* DNA intramuscular injection (IM) (10 μg in 100 μl PBS); (3) 50 μg *ag85a/b* DNA IM (50 μg in 100 μl PBS); (4) 100 μg *ag85a/b* DNA IM (100 μg in 100 μl PBS); (5) 200 μg *ag85a/b* DNA IM (200 μg in 100 μl PBS);(6) 10 μg *ag85a/b* DNA IM +EP (10 μg in 100 μl PBS); (7) 50 μg *ag85a/b* DNA IM +EP (50 μg in 100 μl PBS); (8) 100 μg *ag85a/b* DNA IM +EP (100 μg in 100 μl PBS); (9) 200 μg *ag85a/b* DNA IM +EP (200 μg in 100 μl PBS). On the third day after infection, the mice in groups 1 to 5 were immunized intramuscularly, and in groups 6 to 9 were medicated by EP three times at two-week intervals, respectively. EP was performed using a TERESA Gene Delivery Device (TERESA Health Technology Co., LTD, Shanghai, China), set at 36 Voltage and 25 Hz, and six pulses of 10 ms in 3mm depth of thigh muscle of the hind leg of a mouse.

### Determination of CD4^+^ and CD8^+^ T Cell Subsets Expressing Intracellular IFN-γ or IL-4

The operation procedure was described previously (35). 500 μl of whole blood was stimulated by adding phorbol 12-myristate 13-acetate (PMA, Cat. No. 8139, BD Biosciences, San Jose, Ca, USA) and ionomycin (Cas.10634, Sigma Chemical Co., St.Louis, Mo, USA) as a positive control or by adding 10μl of 1mg/ml Ag85AB protein and then incubating with 5% CO_2_ at 37°C for 4 h in the presence of inhibitor brefeldin A (Cat. No. 347688, BD Biosciences, San Jose, Ca, USA). Samples incubated with brefeldin A alone were used as non-stimulated controls (as a negative control). 200μl of blood was then pipetted directly into a 12 × 75 mm fluorescence-activated cell sorting (FACS) tube containing FITC Hamster Anti-Mouse CD3e (Cat. No. 553061, Clone: 145-2C11, BD Biosciences, San Jose, Ca, USA) and PerCP-Cy™5.5 Rat Anti-Mouse CD8a (Cat. No. 551162, Clone: 53-6.7, BD Biosciences, San Jose, Ca, USA), and incubated at room temperature in the dark for 15 min. Then 60 µl of 1 × permeabilizing solution A (BD IntraSure kit, Cat. No.641776, BD Biosciences, San Jose, Ca, USA) was added into the tube and incubated at room temperature in the dark for 5 min. The erythrocytes were then lysed using 2 ml of 1 × FACS lysing solution (Cat. No. 349202, BD Biosciences, San Jose, Ca, USA) for 12 min before centrifugation at 1500 rpm for 5 min. The supernatant was aspirated and 30 µl of 1 × permeabilizing solution B (BD IntraSure kit, Cat. No.641776, BD Biosciences, San Jose, Ca, USA) and 5 µl of APC Rat Anti-Mouse IFN-γ (Cat. No. 554413, Clone: XMG1.2, BD Biosciences, San Jose, Ca, USA) 5 µl of PE Rat Anti-Mouse IL-4 (Cat. No. 554435, Clone: 11B11, BD Biosciences, San Jose, Ca, USA) were added into the sample tube, 5 µl of APC Rat IgG1 κ isotype control (Cat. No. 554886, Clone: R3-34, BD Biosciences, San Jose, Ca, USA), 5 µl of PE Rat IgG1 κisotype control (Cat. No. 554885, Clone: R3-34, BD Biosciences, San Jose, Ca, USA), respectively, and incubated at room temperature in the dark for 20 min. 1 ml of PBS containing 2% fetal bovine serum (FBS) was added before centrifugation at 1500 rpm for 5 min. The supernatant was aspirated, 500 µl PBS was added, and the cell suspension was transferred into 12 × 75 mm FACS tubes and analyzed within 1 h by flow cytometry. CD4^+^IFN-γ^+^ T cell, CD4^+^IL-4^+^ T cell, CD8^+^IFN-γ^+^ T cell, and CD8^+^IL-4^+^ T cell responding to recombinant Ag85AB proteins were calculated. Cells expressing IFN-γ and IL-4 were presented as a percentage of the total population of CD3^+^ cells. The data were collected using the FACS Calibur flow cytometer (BD Pharmingen) and analyzed using CellQuest software. The representing FACS figures with gating method and representing flow cytometry dot plot were shown in **Supplementary Figure 1**.

### Assay of CD4^+^CD25^+^ Treg and CD4^+^CD25^+^FoxP3^+^ Treg Cell Subsets

The blood (100 µl) with Heparin sodium anticoagulant from seven mice per group was pipetted directly into second 12×75 mm FACS tube containing 2 µl of FITC Rat Anti-Mouse CD4 (Cat. No. 553046, Clone: RM4.5, BD Biosciences, San Jose, Ca, USA) and 5 µl of APC Rat Anti-Mouse CD25 (Cat. No. 557192, Clone: PC61, BD Biosciences, San Jose, Ca, USA) and incubated at room temperature in the dark for 15 minutes. Red blood cells were lysed using 1 ml of 1×FACS lysing solution (Cat. No. 349202, BD Biosciences, San Jose, Ca, USA) for 30 minutes before centrifugation at 1200 rpm for 5 minutes. 1 ml of PBS was added into the tube before centrifugation at 1200 rpm for 5 minutes, the supernatant was aspirated, and 1×fixation/permeabilization concentrate (Foxp3/Transcription factor staining buffer set Cat. No. 00-5523, eBioscience, San Diego, Ca, USA) was added into the tube and incubated for 15 minutes at 4°Cin the dark. 1 ml of PBS was added into the tube before centrifugation at 1200 rpm for 5 minutes, the supernatant was aspirated, and 2 ml of 1× permeabilization buffer (FoxP3/Transcription factor staining buffer set Cat. No. 00-5523, eBioscience, San Diego, Ca, USA)was added into the tube before centrifugation at 1200 rpm for 5 minutes. The supernatant was aspirated, and 2 μl normal rat serum was added into the tube and incubated for 15 minutes at room temperature in the dark. 5 µl of PE Anti-Mouse/Rat FoxP3 (Cat. No. 12-5773, Clone: FJK-16s, eBioscience, San Diego, Ca, USA), 2.5 µl of APC Rat IgG1 λ Isotype control (Cat. No. 550884, BD Biosciences, San Jose, Ca, USA) 2.5 µl of PE Rat IgG2a κ isotype (Cat. No. 12-4321, eBiosciences, San Diego, Ca, USA) were added into the tube and incubated for 40 minutes at 4°C in the dark. 2 ml of permeabilization buffer was added into the tube before centrifugation at 1200 rpm for 5 minutes. The supernatant was aspirated, 300 µl of PBS was added into the tube, and the cell suspension was transferred into 12×75 mm FACS tubes and analyzed in an hour by FACS Calibur flow cytometry (BD Pharmingen) and analyzed using CellQuest software. The FACS figures with gating method and representing flow cytometry dot plot were shown in **Supplementary Figure 2**.

### Bacterium Counts

The mice were sacrificed by cervical dislocation under anesthetic with 5ml dethylether (Beijing Chemical Reagents Company, Beijing, China) at 17-18 days after the third immunization. The tissue suspensions of mouse lungs and spleens were serially diluted 10-fold, and 100 μL suspension dilution was inoculated in duplicate on Lowenstein-Jensen medium plates and cultured at 37°C for four weeks. MTB colonies on the medium were counted, and the results were shown as CFUs per organ.

### Lung Histopathological Examination

The mouse lungs tissues paraffin-embedded were sliced into 3-μm thick tissue sections, which were dyed with hematoxylin and eosin, and then examined by a certified and veteran pathologist.

### Statistical Analyses

Data are shown as means and standard deviations. Statistical analyses were performed using one-way ANOVA followed by Dunnett’s multiple comparison test and two factors factorial design ANOVA followed by compared with t-test using SAS 9.1 software, and a *P*-value of <0.05 was considered to be statistically significant.

## Results

### Immunological Evaluation

**Figure 2:**
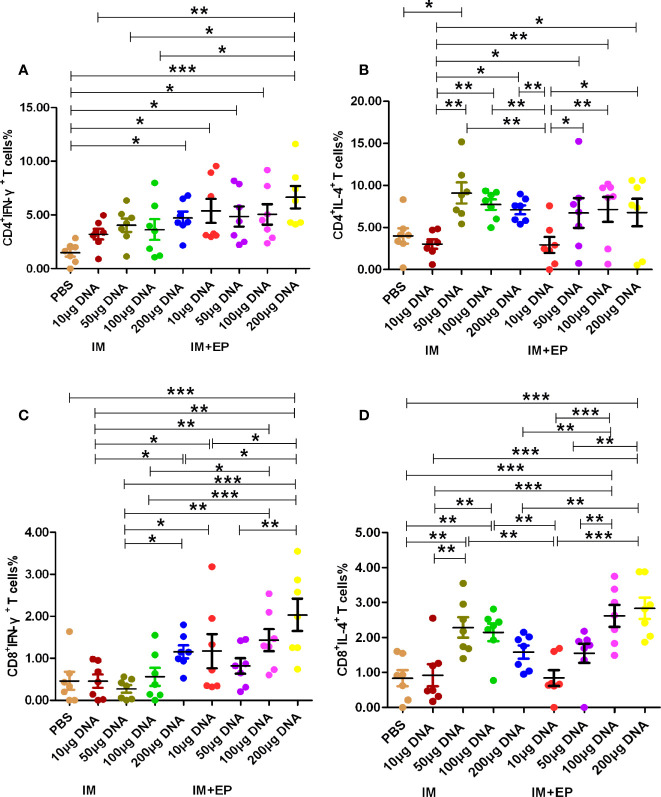
Frequencies of CD4^+^ and CD8^+^ T cell subsets in whole blood were assessed by flow cytometry. At 17-18 days after the third immunization, the data are expressed as the mean% ± standard deviation (n = 7) from 7 mice in each group. **(A)** CD4^+^ T cells expressing IFN-γ (IFN-γ-FITC); **(B)** CD4^+^ T cells expressing IL-4 (IL-4-PE); **(C)** CD8^+^ T cells expressing IFN-γ (IFN-γ-FITC); **(D)** CD8^+^ T cells expressing IL-4 (IL-4-PE). IM, intramuscular injection; EP, electroporation. *P<0.05, **P < 0.01, ***P < 0.0001.

**Figure 3:**
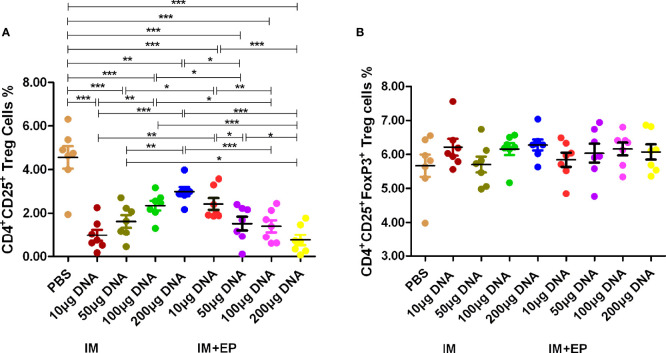
Frequencies of CD4^+^ and CD25^+^ Treg cell subsets in whole blood were assessed by flow cytometry. At 17-18 days after the third immunization, the data are expressed as the mean% ± standard deviation (n = 7) from 7 mice in each group. **(A)** Frequencies of CD4^+^ CD25^+^ Treg cells; **(B)** Frequencies of CD4^+^CD25^+^Foxp3^+^ Treg cells. IM, intramuscular injection; EP, electroporation. *P < 0.05, **P < 0.01, ***P < 0.0001.

The results of immunological evaluation on mouse TB model immunized with different doses of MTB *ag85a/b* DNA vaccine by IM and EP were shown in **Figures 2**, **3**. The proportion of CD4^+^ T cells expressing IFN-γ in response to recombinant Ag85AB chimeric protein in the whole blood from all DNA groups were higher than that in the PBS group by flow cytometry, but there was a significant difference among those only in the 200 μg DNA IM group and 10 μg, 50 μg, 100 μg, 200 μg DNA EP groups (*P*<0.05, *P*<0.0001, **Figure 2A**). There was no significant difference among those in the four doses of DNA IM groups or the DNA EP group (*P*>0.05). The proportion of CD4^+^ T cells expressing IL-4 in the blood only from the 50μg DNA IM group was significantly higher than that of the PBS group (*P*<0.05, **Figure 2B**).

The proportion of CD8^+^ T cells expressing IFN-γ in response to recombinant Ag85AB chimeric protein in the whole blood from all DNA groups were higher than that of the PBS group, but only the 200 μg DNA EP group was significantly higher than that of the PBS group (*P*<0.01, **Figure 2C**). The proportion of CD8^+^IFN-γ^+^ T cells in the 10μg, or 100 μg or 200 μg DNA EP group was significantly higher than that in the 10 μg or 100 μg or 200 μg DNA IM group (*P*<0.05), the proportion of CD8^+^IFN-γ^+^ T cells in the 50μg DNA EP group was higher than that in the 50 μg DNA IM group, but there was no significant difference among them (*P*>0.05). The proportion of CD8^+^ T cells expressing IL-4 in the blood from the 50 μg, 100 μg DNA IM groups and 100 μg, 200 μg DNA EP groups were significantly higher than that in the PBS group (*P*<0.01, *P*<0.0001, **Figure 2D**). The proportion of CD8^+^IL-4^+^ T cells in the 50μg and 100 μg DNA IM group was significantly higher than those in the 10 μg DNA IM group and 10 μg DNA EP groups (*P*<0.01, *P*<0.0001). The proportion of CD8^+^IL-4^+^ T cells in the 100 μg and 200 μg DNA EP group was higher than that in the 10 μg,and 200 μg DNA IM, 10μg and 50μg DNA EP groups (*P*<0.01, *P*<0.0001), but there was no significant difference between them (*P*>0.05).

The proportion of CD4^+^CD25^+^ Treg cells in the whole blood from all DNA groups was significantly lower than that of the PBS group by flow cytometry (*P*<0.01, *P*<0.0001, **Figure 3A**), but there was no significant difference in the proportion of CD4^+^CD25^+^ FoxP3^+^ Treg of all groups (*P*>0.05, **Figure 3B**). The proportion of CD4^+^CD25^+^ Treg cells in the 10 μg DNA EP group was higher than that in the 10 μg DNA IM group (*P*<0.001); the proportion of CD4^+^CD25^+^ Treg cells in the 100 μg and 200 μg DNA EP group were significantly lower than those in the same doses of DNA IM group (*P*<0.05, *P*<0.0001). The proportion of CD4^+^CD25^+^ Treg cells in the 100 μg DNA IM group was significantly higher than those in the 10 μg DNA IM group and 50 μg, 100 μg, 200 μg DNA EP groups (*P*<0.05, *P*<0.001, *P*<0.0001), but was lower than that in the 200 μg DNA IM group (*P*>0.05). The proportion of CD4^+^CD25^+^ Treg cells in the 50 μg DNA EP group was significantly lower than those in the 10 μg DNA EP group and 100 μg, 200 μg DNA IM groups (*P*<0.05, *P*<0.001), but was significantly higher than that in the 200 μg DNA EP group (*P*<0.05).

### Bacterial Counts in the Lungs and Spleens

**Figure 4:**
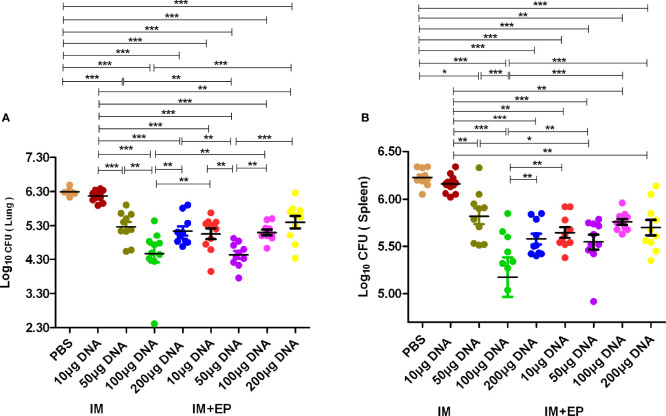
The numbers of live bacteria in lungs **(A)** and spleens **(B)** at 17-18days after the third immunization. The data are expressed as the mean% ± standard deviation (n = 10) from 10 mice in each group. IM, intramuscular injection; EP, electroporation. *P < 0.05, **P < 0.01, ***P < 0.0001.

The live bacteria in mouse lungs and spleens were determined at 17-18 days after the third immunotherapy (shown in **Figure 4**). Compared with the PBS group, except the 10 μg DNA IM. group, the CFUs of mouse lungs and spleens in other doses of DNA vaccine groups delivered by IM and EP decreased significantly (*P*<0.0001, *P*<0.01, *P*<0.05). The CFUs of mouse lungs and spleens in10 μg and 50 μg DNA EP groups were significantly lower than those in the same doses of the DNA IM group (*P*<0.0001, *P*<0.01, *P*<0.05). However, the CFUs of mouse lungs and spleens in the 100μg DNA EP group was higher than that in the 100 μg DNA IM. group (*P*< 0.01, *P*< 0.0001), and in the 200μg DNA EP group were slightly higher than that in the 200 μg DNA IM group, but there was no significant difference (*P*>0.05). There was no significant difference in lung CFUs between the 100 μg DNA IM group and the 50μg DNA EP group (*P*>0.05). These two groups were significantly lower than those in other groups (*P*<0.01, *P*<0.0001). The spleen CFU in the 50μg DNA EP group was significantly higher than that in the 100 μg DNA IM group (*P*<0.01), but lower than other groups, especially significantly lower than the 10 μg and 50 μg DNA IM group (*P*<0.05, *P*<0.0001). As a result, the 100 μg DNA IM group and 50 μg DNA EP group had a better therapeutic effect on the mouse TB model.

### Histopathological Changes

**Figure 5:**
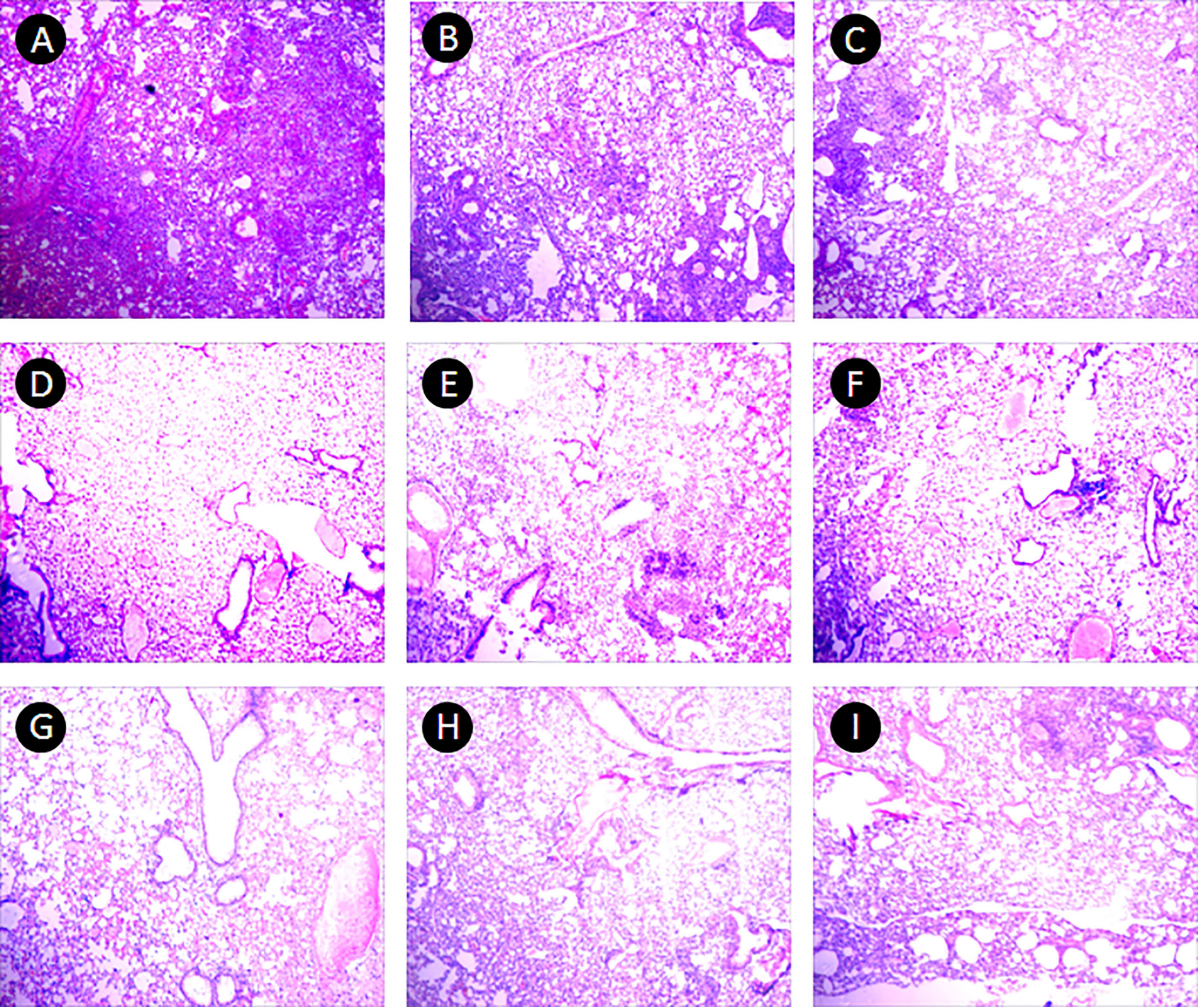
Pulmonary histopathological changes. This figure shows representative photomicrographs (H and E, 40×) of lung tissue obtained from mice each group at 17-18 days after the third immunization. **(A)** PBS group. **(B–E)**, 10 μg, 50 μg, 100 μg and 200 μg *ag85a/b* DNA IM groups. **(F–I)**, 10 μg, 50 μg, 100 μg and 200 μg *ag85a/b* DNA EP groups. IM, intramuscular injection; EP, electroporation.

In the PBS group, the alveolar wall was severely thickened, a large number of lymphocytes infiltrated under the bronchioles mucosa, and large granuloma nodules were formed locally, its percentage of lung lesion area was (65 ± 23)%. In the 10 μ g DNA IM group, the lesions were similar to that in the PBS group, and more granuloma nodules were formed locally, its percentage of lung lesion area was (58 ± 28)%. In the 50 μg, 200 μg DNA IM group, and in the 10 μ g, 100 μg, 200 μg DNA EP groups, the lesions were lighter, the alveolar wall was slightly thickened, small granulomatous nodules were formed locally, with a small amount of lymphocyte infiltration, and the area of the lesions was reduced at different agree, their percentages of lung lesion area were (54 ± 27)%, (52 ± 25)%, (51 ± 27)%, (52 ± 23)% and (55 ± 27)%, respectively. The lesions in the 100 μg DNA IM group and 50 μg DNA EP group were the lightest, and most of the alveolar was relatively clear and had typical structures, their percentage of lung lesion areas were perspectively (39 ± 25)% and (39 ± 33)%, which were significantly lower than that in the PBS group (*P*<0.05). Representative histopathological changes of nine groups were shown in **Figure 5**.

## Discussion

EP of muscle has proven to be efficient in enhancing gene delivery, transgene expression, and the immunogenicity of DNA vaccines encoding antigens in both small and large animals (36). More plasmids were delivered into the muscle cells by EP, and then antigen expression in muscle is improved 100- to 1000-fold compared to antigen expression from intracellular uptake of plasmids by naked DNA injection (37). Zucchelli et al. (38) constructed a DNA vaccine encoding HCV E2 glycoprotein, the gene expression increased by 10 times in mouse tissues and antibody levels increased 10-30 times in BALB/c mice, CD rats, and New Zealand rabbits by EP, respectively. Li Dingfeng et al. (39) applied EP technology to enhance the immunogenicity of HIV Gag DNA, the specific antibody levels increased by 28 times, but the cellular immune response did not enhance. Anti-tuberculosis immunity is primarily a cell-mediated immune response. The combination of suitable novel prominent TB antigens was incorporated into DNA vaccines could drive both a CD4 and CD8 T cell-mediated immune response, which has emerged as a promising approach (40). Therefore, in this study, we compared the immunotherapeutic of different doses of MTB *ag85a/b* chimeric DNA vaccine delivered by intramuscular injection and EP technology on the mouse TB model, to improve DNA immunogenicity, reduce DNA dosage, and achieve the same therapeutic effect by EP.

Inducing a strong Th1-type immune response can induce anti-TB protection. Inhibition of Th2-type immune response can also produce beneficial intervention effects (41, 42). Therefore, the detection of Th1-/Th2-type immune responses and their cytokines has become an important evaluation index for the immunogenicity of the TB vaccine. Th1-type cells produce cytokines such as IFN-γ. IFN-γ is mainly produced by activated CD4^+^, CD8^+^ T cells, and natural killer cell, and play a wide range of anti-TB immune effects. Th2-type cells mainly secrete cytokines such as IL-4. IL-4 can inhibit the proliferation and differentiation of Th1 cells and induce a humoral immune response. Treg cells are a subset of T cells that express CD4 and CD25 molecules. CD4^+^CD25^+^ Treg cells account for 5% - 10% of CD4^+^ T cells. Its main function is to inhibit the activation of other effector T cells, induce immune tolerance and play an immunomodulatory role (43). Foxp3 T cells are a subset of CD4^+^CD25^+^ Treg cells, express CD45RO subtype, GITC and CD152 molecules on the surface, and Foxp3 transcription factor in the nucleus. CD4^+^CD25^+^Foxp3^+^ Treg cells can inhibit anti-TB immune function or release anti-inflammatory factors IL-10 and TGF-β,effectively down-regulate Th1-typeimmune function and promote the latency and proliferation of MTB (44, 45). Tollefsen S et al. immunized mice with 120 µg *ag85a* DNA, 100 µg PAP lacZ DNA and 100 µg *ag85b* DNA by electroporation, the number of antigen-specific CD4^+^ and CD8^+^ T cells detected by ELISPOT assay increased 3 to 6 times compared with the mice without electroporation (46). In this study, compared with the PBS group, the CD4^+^IFN-γ^+^ T cells% in 200 µg DNA IM group and four DNA EP groups increased significantly (*P*< 0.05), in 10 µg, 50 µg and 100 µg DNA IM group also higher than that in the PBS group, but there was no significant difference; the CD4^+^IFN-γ^+^ T cells% in four DNA EP groups were higher than those of four DNA IM groups. The detection of flow cytometry in this study used comparative counting method, the CD4^+^IFN-γ^+^ T cells% increased in DNA vaccine groups, so that CD8^+^IFN-γ^+^ T cells% (in 200 μg DNA EP group), CD4^+^IL-4^+^ T cells% (50μg DNA IM group) and CD8^+^IL-4^+^ T cells% (50 μg and 100 μg DNA IM groups, 100 μg and 200 μg DNA EP groups) increased significantly only in a few DNA groups (*P*< 0.05).The CD4^+^CD25^+^ Treg cells% decreased significantly in all DNA vaccine groups, and CD4^+^CD25^+^ Foxp3^+^ Treg cells had no significant difference. These results suggest that MTB *ag85a/b* chimeric DNA vaccines a strong inducer of CD4^+^IFN-γ^+^ T cells, and EP could further enhance Th1-type immune response. Treg cells decreased or remained unchanged, indicating the ratio between these two types of cells can balance protection and lung injury (30, 47). Therefore, EP immunization can enhance the immune response of low-dose DNA vaccines, and a small amount of DNA vaccine can produce strong immunogenicity.

The bacterial organ load is one of the essential indicators to evaluate curative effects on animal TB experiments (48). Compared with the PBS group, the lung and spleen CFUs of the other seven DNA immunization groups except for the 10 μg DNA IM group decreased significantly, especially 50 μg DNA EP group and 100 μg DNA IM group significantly reduced the pulmonary bacterial loads than the other DNA groups, which were consistent with its lightest lung lesions. Similarly, Sallberg et al. reported a transient reduction in viral load (0.6 log_10_ to 2.4 log_10_) in patients infected with chronic hepatitis C vaccinated with an NS3/4a based DNA vaccine delivered *via* EP (49). There were dose-dependent effects in the 10 μg, 50 μg, and 100 μg IM groups, 10 μg and 50 μg DNA EP groups, which is in agreement with our previous studies (29, 30, 50). The appropriate amount of DNA delivered by IM or EP can express sufficient protein *in vivo* to induce an effective cellular immune response and protect mice against MTB invasion. Compared with the antigen expression of plasmid ingested by naked DNA injection, EP can deliver more plasmids to muscle cells and improve the antigen expression in muscle. The less DNA delivered by EP can achieve the immunotherapeutic effect of 100 μg DNA IM on the mouse TB model. Delivering too much DNA would induce a Th2-type immune response. For example, the effect of the 100 μg DNA EP group was significantly lower than the 50 μg DNA EP group, which is consistent with our previous studies (51). Overall, the protective immune response induced was consistent with the bacterial lung load and lesion range. These results suggest that DNA immunization by EP could improve the immunogenicity of the low-dose DNA groups, which is lower than the effective dose of intramuscular injection, reduce the number of bacteria and the range of lesions, so that the 50 μg DNA EP group could achieve the immunotherapeutic effects of the 100 DNA IM group on the mouse TB model. The clinical trials from Yan J et al. showed that the main adverse effect (grade 1/2) related to the EP procedure was temporary pain, which quickly subsided within 25-30 minutes (52), indicating that EP had no apparent side effects. The characteristics of EP to improve immunogenicity may lead to the progress of delivery technology. When DNA vaccine is applied to larger animals or humans in the future, it is essential to determine the optimal DNA dose.

## Conclusion

An MTB *ag85a/b* chimeric DNA vaccine could induce Th1-type cellular immune reactions. DNA immunization by EP could improve the DNA immunogenicity of the low-dose DNA vaccine, reduce DNA dose, produce sound immunotherapeutic effects on TB, and provide the basis for the future human clinical trial of MTB *ag85a/b* chimeric DNA vaccine.

## Data Availability Statement

The original contributions presented in the study are included in the article/**Supplementary Material**. Further inquiries can be directed to the corresponding author.

## Ethics Statement

The animal study was reviewed and approved by The Animal Ethical Committee of the 8th Medical Center of the Chinese PLA General Hospital.

## Author Contributions

Conceptualization: XW and YL. Methodology: YL, LC, LX, XL, YY, YBL, TW, LW, and JW. Data analysis: YL. Wrote original manuscript: YL. Review and revise manuscript: XW and YL. All authors contributed to the article and approved the submitted version.

## Funding

This project was supported by the grant from the Serious Infectious Diseases Special Foundation (2012ZX10003008-002, 2018ZX10731301-005), the Special Key Project of the Medical Innovation Project of China (18CXZ028).

## Conflict of Interest

The authors declare that the research was conducted in the absence of any commercial or financial relationships that could be construed as a potential conflict of interest.

## Publisher’s Note

All claims expressed in this article are solely those of the authors and do not necessarily represent those of their affiliated organizations, or those of the publisher, the editors and the reviewers. Any product that may be evaluated in this article, or claim that may be made by its manufacturer, is not guaranteed or endorsed by the publisher.
