# Supplementary material for: Immunotherapeutic Effects of Different Doses of Mycobacterium tuberculosis ag85a/b DNA Vaccine Delivered by Electroporation
Source: Front Immunol. 2022 May 4;13:876579. doi: 10.3389/fimmu.2022.876579 (PMC9114437; doi:10.3389/fimmu.2022.876579)
Supplement: Supplementary file 1 [file DataSheet_1.pdf]

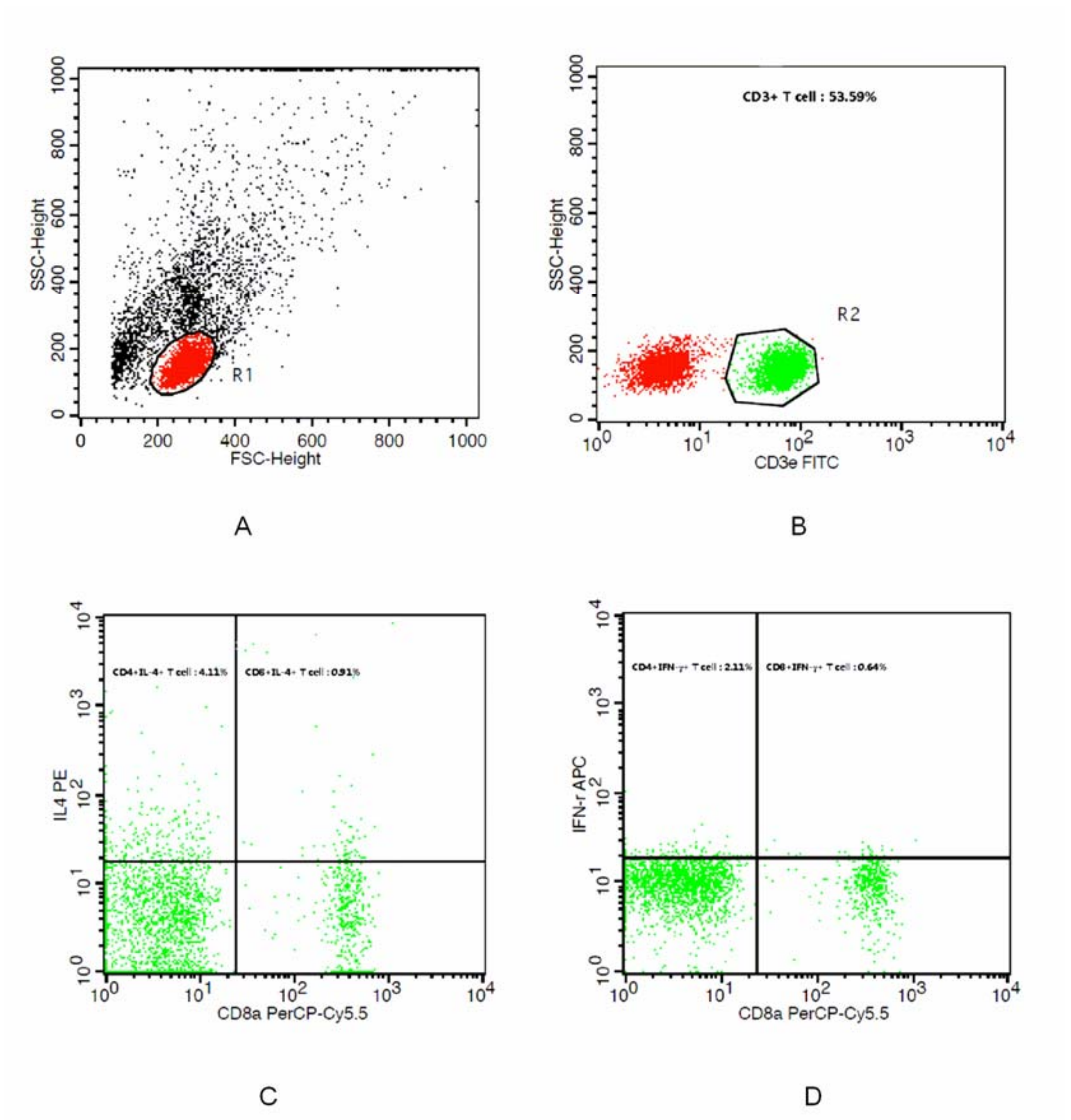

Supplementary figure 1 Flow cytometry analysis of CD3$^+$ T cells gated on single cells(**A**)**(B)** , and CD4$^+$IFNɣ$^+$ T cell **(Up Left of D)**, CD4$^+$IL-4$^+$ T cell **(Up Left of C)**, CD8$^+$IFNɣ$^+$ T cell **(Up Right of D)**, CD8$^+$ IL-4$^+$ T cells **(Up Right of C)**（C and D gated on CD3$^+$T cells）**.** CD4$^+$IFNɣ$^+$ T cell / CD4$^+$IL-4$^+$ T cell / CD8$^+$IFNɣ$^+$ T cell /Tc2 CD8$^+$ IL-4$^+$ T cells shown is the result of removing the background.
