# Supplementary material for: Immunotherapeutic Effects of Different Doses of Mycobacterium tuberculosis ag85a/b DNA Vaccine Delivered by Electroporation
Source: Front Immunol. 2022 May 4;13:876579. doi: 10.3389/fimmu.2022.876579 (PMC9114437; doi:10.3389/fimmu.2022.876579)
Supplement: Supplementary file 2 [file DataSheet_2.pdf]

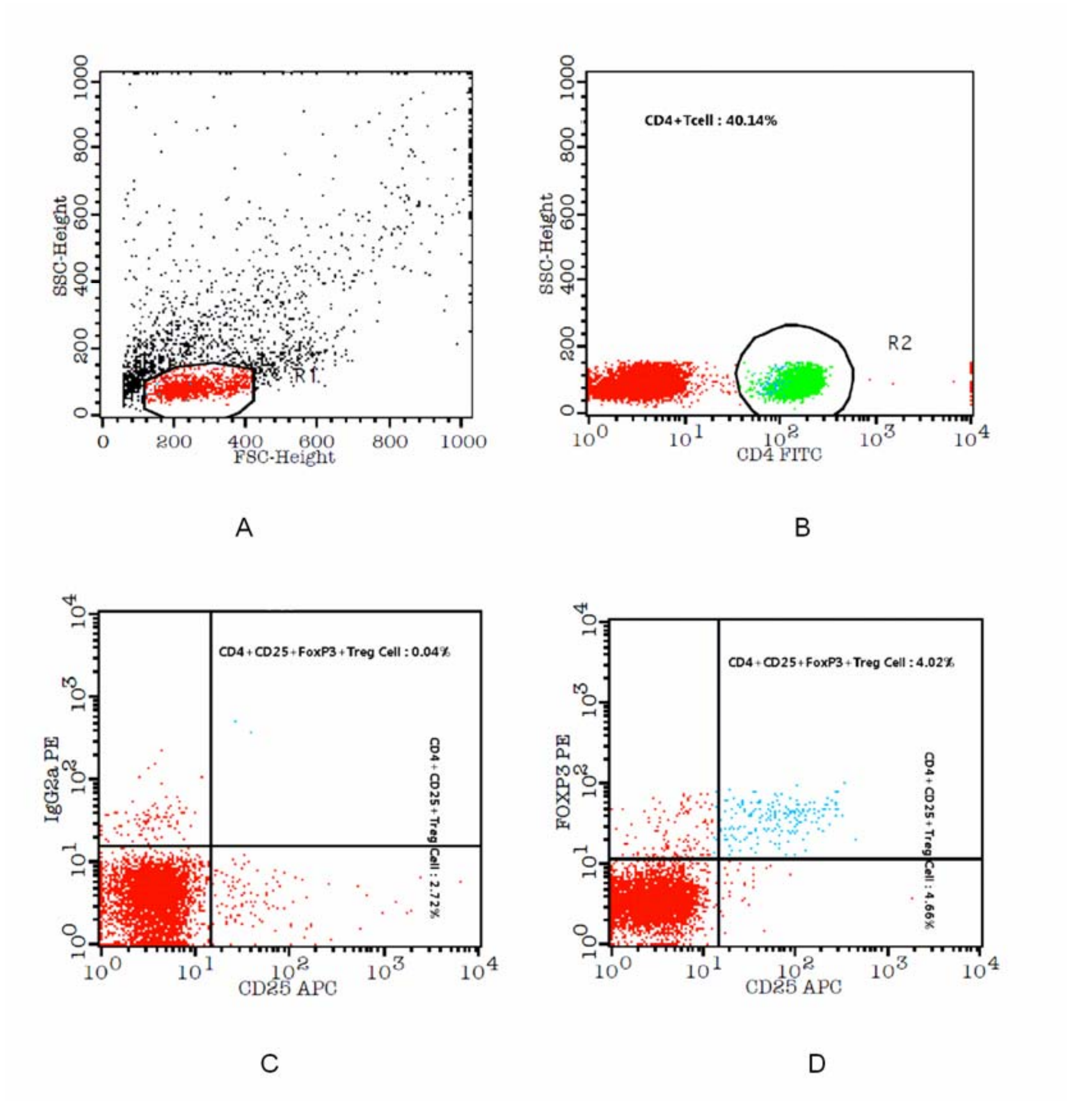

Supplementary figure 2 Flow cytometry analysis of $CD4^+$ T cells gated on single cells (**A**)**(B),** and $CD4^+CD25^+$ Treg Cell **(Right of D)**, $CD4^+CD25^+FoxP3^+$ Treg Cell **(Up Right of D)**, (C and D gated on $CD4^+$ T cells）, final result of $CD4^+CD25^+FoxP3^+$ Treg Cell and $CD4^+CD25^+$ Treg Cell had removed the background of homotypic antibody **(C).**
